# ***De novo* genome assembly and annotation of Australia's largest freshwater fish, the Murray cod (*Maccullochella peelii*), from Illumina and Nanopore sequencing read**

**DOI:** 10.1093/gigascience/gix063

**Published:** 2017-07-19

**Authors:** Christopher M. Austin, Mun Hua Tan, Katherine A. Harrisson, Yin Peng Lee, Laurence J. Croft, Paul Sunnucks, Alexandra Pavlova, Han Ming Gan

**Affiliations:** 1 Centre for Integrative Ecology, School of Life and Environmental Sciences, Deakin University, Geelong, Victoria 3220, Australia; 2 Genomics Facility, Tropical Medicine and Biology Platform, Monash University Malaysia, Jalan Lagoon Selatan, Bandar Sunway 47500, Petaling Jaya, Selangor, Malaysia; 3 School of Science, Monash University Malaysia, Jalan Lagoon Selatan, Bandar Sunway 47500, Petaling Jaya, Selangor, Malaysia; 4 School of Biological Sciences, Monash University, Clayton Campus, Clayton, Victoria, Australia; 5 Malaysian Genomics Resource Centre Berhad, Boulevard Signature Office, Kuala Lumpur, Malaysia

**Keywords:** Murray cod, long reads, genome, transcriptome, hybrid assembly

## Abstract

One of the most iconic Australian fish is the Murray cod, *Maccullochella peelii* (Mitchell 1838), a freshwater species that can grow to ∼1.8 metres in length and live to age ≥48 years. The Murray cod is of a conservation concern as a result of strong population contractions, but it is also popular for recreational fishing and is of growing aquaculture interest. In this study, we report the whole genome sequence of the Murray cod to support ongoing population genetics, conservation, and management research, as well as to better understand the evolutionary ecology and history of the species. A draft Murray cod genome of 633 Mbp (N_50_ = 109 974bp; BUSCO and CEGMA completeness of 94.2% and 91.9%, respectively) with an estimated 148 Mbp of putative repetitive sequences was assembled from the combined sequencing data of 2 fish individuals with an identical maternal lineage; 47.2 Gb of Illumina HiSeq data and 804 Mb of Nanopore data were generated from the first individual while 23.2 Gb of Illumina MiSeq data were generated from the second individual. The inclusion of Nanopore reads for scaffolding followed by subsequent gap-closing using Illumina data led to a 29% reduction in the number of scaffolds and a 55% and 54% increase in the scaffold and contig N_50_, respectively. We also report the first transcriptome of Murray cod that was subsequently used to annotate the Murray cod genome, leading to the identification of 26 539 protein-coding genes. We present the whole genome of the Murray cod and anticipate this will be a catalyst for a range of genetic, genomic, and phylogenetic studies of the Murray cod and more generally other fish species of the *Percichthydae* family.

## Data Description

**Figure 1:**
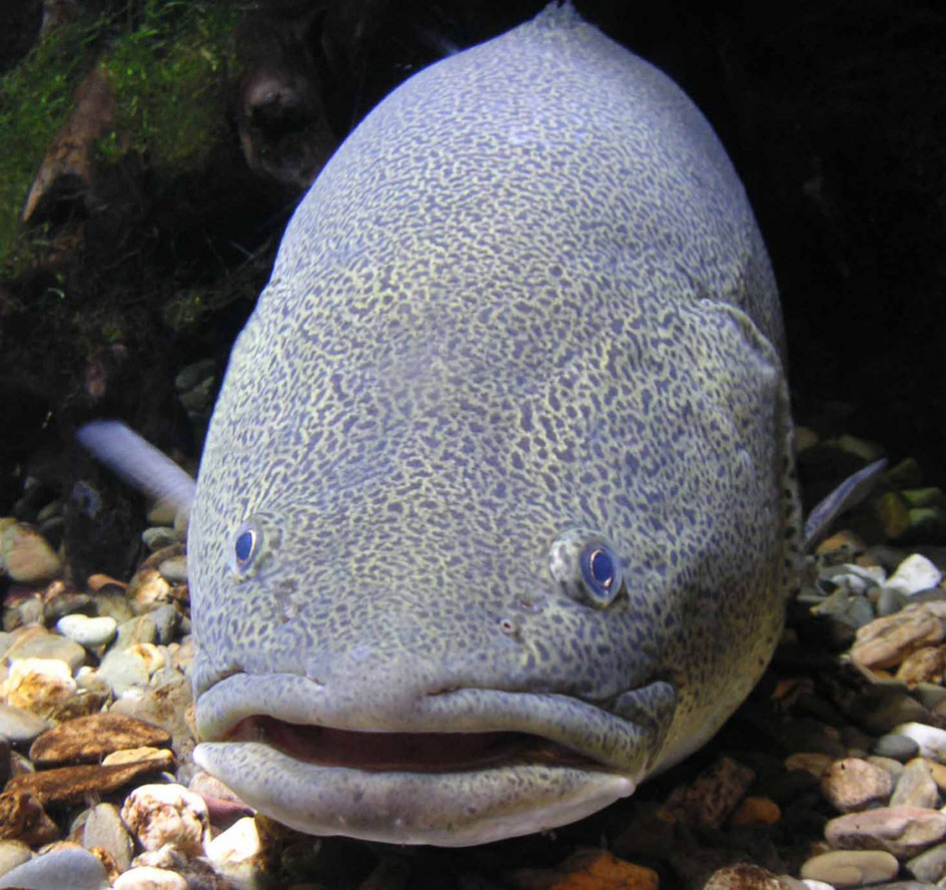
The iconic Murray cod. Photo: Paul Sunnucks.

Population genetic and evolutionary studies on Australian freshwater fish are of special interest in relation to conservation, biogeography, and adaptive responses and have been studied using a range of molecular techniques [1–8]. A limitation to a more complete understanding of the genetics and evolution of Australian inland fish species is the lack of genome-level resources [9]. The Murray cod, *Maccullochella peelii* (NCBI Taxon ID: 135761, Fishbase ID: 10311), is one of Australia's most iconic large (up to ∼1.8 metres) and long-lived (≥48 years) predatory fish species that occurs across highly variable and heterogeneous riverine environments of inland Australia (Fig. 1). Despite being widespread, the Murray cod is a threatened species under national legislation (Environment Protection and Biodiversity Conservation Act 1999), and populations are intensively managed through programs such as habitat restoration, provision of environmental flows, and stocking.

## Sampling, Library Construction, and Sequencing

Sequencing data from 2 Murray cod individuals were merged for whole genome assembly. The first individual was obtained from an Australian fish market in 2014 [5]. Genomic DNA was extracted from multiple fin clip and muscle samples using DNAeasy Blood and Tissue Kits (Qiagen, Halden, Germany). A 300-bp insert library was prepared from the purified gDNA using the TruSeq DNA sample prep kit (Illumina, San Diego, CA, USA) according to the manufacturer's instructions and subsequently sequenced (2 × 100 bp, 1 × 100 bp configurations) on a HiSeq 2000 (Illumina, San Diego, CA, USA), located at the Malaysian Genomics Resource Centre Berhad. For sequencing on the MinION, gDNA was extracted from the remaining fin clip and muscle tissues that were collected in 2014. However, due to DNA degradation associated with long-term storage, an additional size selection (8–30 kb) with a BluePippin was performed to reduce the representation of short reads (Sage Science, Beverly, MA, USA). Seven individual libraries (2 1D preps and 5 2D preps) were prepared and sequenced on 7 R9 flowcells using the MinION portable DNA sequencer (Oxford Nanopore, Oxford, UK) according to the manufacturer's instructions. The second Murray cod, isolate KMC200 (=MCC0324) [2], was sampled from the Lachlan River in New South Wales in 2006, and its library has been previously constructed and sequenced at the Monash University Malaysia Genomics Facility for a mitogenome-based population genetics study [2, 5]. Given that the whole mitogenome of isolate KMC200 (= MCC0324; GenBank accession number: KT337332.1) exhibits a 100% nucleotide identity to that of the first individual (GenBank accession number: NC_023807.1), indicating a recently shared maternal ancestry [2, 5], its remaining library was re-sequenced on 3 separate MiSeq runs (2 × 250 bp configuration) to improve the sequencing coverage of the Murray cod genome. A total of 70.6 Gb (47.4 Gb and 23.2 Gb from HiSeq and MiSeq runs, respectively) and 804 Mb (N_50_: 4438 bp, longest read: 129 945 bp) of nucleotide sequence were generated on the Illumina platforms and the Oxford Nanopore MinION device, respectively.

## Genome Characteristics

**Figure 2:**
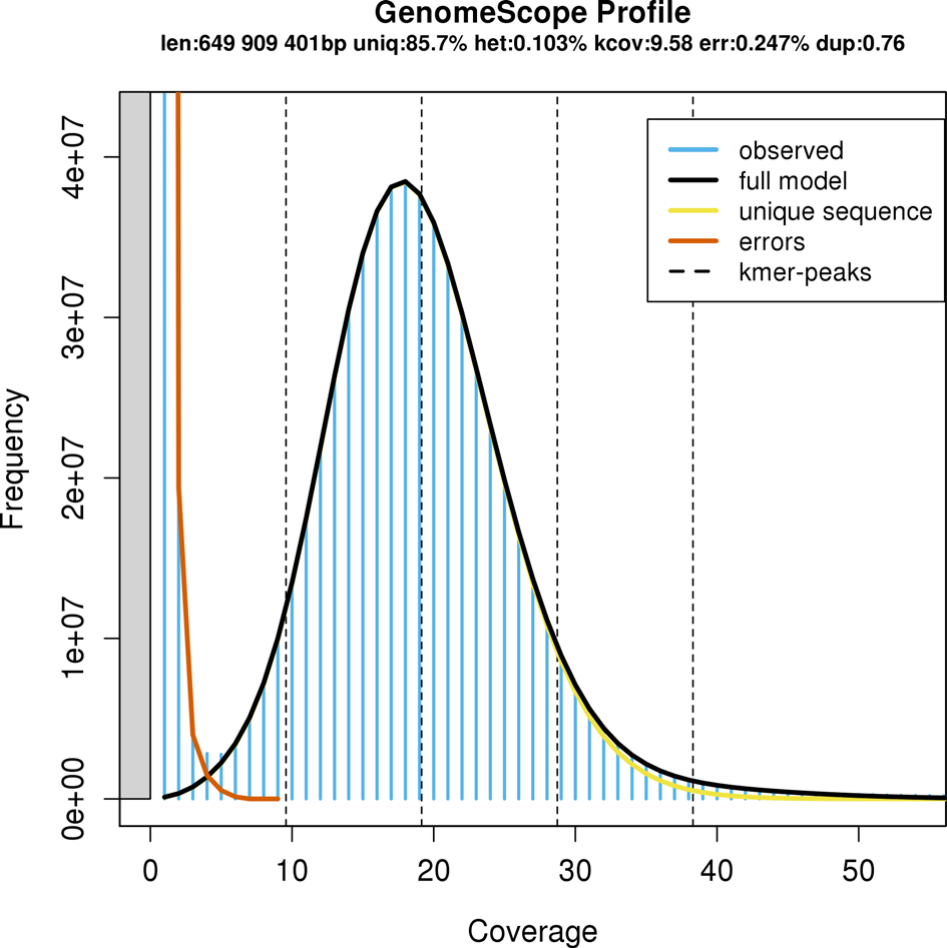
Estimation of genome size, repeat content, and heterozygosity by GenomeScope, based on 21-mers in HiSeq sequence reads (max kmer coverage at 1000).

Jellyfish v. 2.2.6 [10] was used to obtain a frequency distribution of 17-, 21-, 25-, and 31-mers in a subset (∼20 Gb) of the raw HiSeq sequence reads, and the histograms were uploaded to GenomeScope for estimation of genome size, repeat content, and heterozygosity, based on a kmer-based statistical approach [11]. The resulting analysis shows that the haploid genome size was between 640 and 669 Mbp for the Murray cod (Fig. 2), a figure smaller than the 812 Mbp (C-value: 0.83 pg) estimated size reported on the Animal Genome Size Database [12, 13]. This smaller estimate may be due to an additional parameter introduced in GenomeScope, set to exclude extremely high-frequency kmers as these likely represent organelle sequences or other contaminants that can inflate the genome size [11]. Further, the 21-mer analysis (with “max kmer coverage” set at 1000) on GenomeScope also indicates 14.3% repeat content and a low heterozygosity of 0.103%. To test that both Murray cod isolates possess the same genome characteristics, the Jellyfish and GenomeScope analysis was repeated for 23 Gb of MiSeq sequence reads, which resulted in comparable results (643 to 673 Mbp haploid genome size, 15.7% repeat content, a low heterozygosity of 0.113%) (Supplementary Fig. S1; for combined data set, see Supplementary Fig. S2). Further repeat-content analysis and masking is performed in subsequent sections in this study (see “Repeat-content analysis”).

## Genome Assembly

Illumina reads were trimmed with *platanus_trim* v. 1.0.7 (*-q* 20, *-l* 35) and assembled with the Platanus v. 1.2.4 assembler to account for a potential increase in genome heterozygosity due to the use of sequencing data from 2 individuals with shared maternal ancestry [14]. The initial assembly is 622 Mb in length, comprising 80 098 scaffolds with an N_50_ of 68 937 bp (Table 1). The assembly was subsequently scaffolded with SSPACE-LongRead v. 1–1 (BLASR aligner, default settings, minimum 3 links [long reads] required for scaffolding) [15] using long-read MinION sequences (0.93 × coverage), which was base-called offline with Albacore/ONT Sequencing Pipeline Software v. 0.7.4 followed by further gap-filling with Illumina reads using GapFiller v. 1–10 [16].

**Table 1:** Murray cod assembly and annotation statistics

Genome assembly	Illumina only	Illumina (≥500 bp)	Illumina + Nanopore (≥500 bp)
Number of contigs	95 612	41 152	45 882
Contig N_50_ size	33 442 bp	34 269 bp	52 687 bp
Longest contig	328 477 bp	328 477 bp	501 239 bp
Number of scaffolds	80 098	25 642	18 198
Total scaffold size	622 421 194 bp	609 090 121 bp	633 241 041 bp
Scaffold N_50_ size	68 937 bp	70 993 bp	109 974 bp
Longest scaffold	548 726 bp	548 726 bp	1 119 190 bp
% GC/AT/N	40.7/59.1/0.2	40.7/59.2/0.1	40.4/58.7/0.9
CEGMA completeness	89.52%	84.68%	91.94%
Complete BUSCOs	4228 (92.3%)	4229 (92.3%)	4317 (94.2%)
Complete and single-copy BUSCOs	4115 (89.8%)	4115 (89.8%)	4202 (91.7%)
Complete and duplicated BUSCOs	113 (2.5%)	114 (2.5%)	115 (2.5%)
Fragmented BUSCOs	224 (4.9%)	222 (4.8%)	156 (3.4%)
Missing BUSCOs	132 (2.8%)	133 (2.9%)	111 (2.4%)
Transcriptome assembly			
Number of transcripts	321 855		
Transcriptome size	305 149 376 bp		
Mean transcript length	948.10 bp		
Longest transcript	23 655 bp		
CEGMA completeness	99.19%		
Annotation			
Number of protein-coding genes	26 539		
Mean gene length	10 115.3 bp		
Longest gene	134 909 bp		
With functional annotation	25 607		

By adding only 804 Mb of Nanopore reads, we observed improvements in the final 633 Mb assembly, reducing the number of scaffolds (≥500 bp) by 29%, from 25 642 to 18 198, and increasing the scaffold N_50_ by 55%, from 70 993 bp to 109 975 bp. In addition, based on results from read alignment performed with Bowtie2 v. 2.3.2 [17], only a small percentage of the scaffolds representing less than <0.005% of the total assembly size were unique to 1 donor (Supplementary Data 1).

Genome completeness was estimated using 2 separate programs, CEGMA (CEGMA, RRID:SCR_015055) and BUSCO v. 3.0 (BUSCO, RRID:SCR_015008). For BUSCO analysis (-m geno –sp zebrafish settings), the genome was searched against the actinopterygii database (actinopterygii_odb9), which was constructed from 20 fish species consisting of 4584 orthologs. Final genome completeness percentages of 94.2% and 91.94% were estimated by BUSCO and CEGMA, respectively. Further, both analyses also indicate a slight improvement in the genome completeness with the inclusion of Nanopore reads for scaffolding and subsequent gap-closing using Illumina short reads. The small amount of available Nanopore long reads in this study resulted in a limitation in the program of choice in assembly as well as scaffolding. At the time of this study, we chose to use SSPACE-LongRead [15] as a scaffolder as it has been used in several genome assembly publications utilizing Nanopore reads, albeit mostly bacterial genome assemblies [18, 19], reviews [20–22], and benchmarking studies [23–26], as well as some eukaryotic genome assemblies that utilized BAC or fosmid libraries or PacBio long read data [27–29]. While no formal testing on eukaryotic genomes and Nanopore long reads was done by Boetzer and Pirovano [15] in their publication, there is mention of the potential of the method applied on Nanopore reads and eukaryotic assemblies. We have found SSPACE-LongRead to be effective in the scaffolding of the Murray cod contigs as elaborated earlier and also in Table 1. Though the gene content appears to support the validity of the assembly, this study does not include further assessment or verification of the accuracy of the scaffold extensions by SSPACE-LongRead [15]. It is noteworthy, however, that a greater range of assembly and scaffolder programs has become available for large eukaryotic genomes; these programs are worth exploring in future studies [24, 26, 30–32].

## Repeat-Content Analysis

To identify repeats in the assembly, a *de novo* repeat library was first built with RepeatModeler v. 1.0.4 (RepeatModeler, RRID:SCR_015027) [33] using default parameters based on the larger scaffolds (≥5 kb) in the assembly. RepeatMasker v. open-4.0.7 (RepeatMasker, RRID:SCR_012954) [34] was then used to align sequences from the whole assembly to the RepeatMasker Combined Library (Dfam_Consensus 20 170 127 [35] and RepBase 20 170 127 [36]) as well as the *de novo* repeat library to screen for repeats and low-complexity sequences in the assembly. Repeat sequences were estimated to account for 23.38% (148 Mb) of the Murray cod assembly presented in this study.

## Transcriptome Assembly

Total RNA was extracted using the RiboPure RNA purification Kit (Thermo Fisher Scientific, Waltham, MA, USA) from the liver, brain, and muscle tissues of a juvenile Murray cod that was collected from a natural population in Broken Creek under a DELWP collecting permit and euthanized using approved procedures under a Monash ethics permit (BSCI/2012/19). Thirty μL of 300 ng/μL of each RNA extract was pooled and processed as a single sample using the TruSeq RNA library kit (Illumina, San Diego, CA, USA) to generate a 160-bp insert size library. The library was subsequently sequenced on 1 lane of HiSeq2000 (2 × 100 bp configuration) at the Ramaciotti Centre for Gene Function Analysis. A total of 376 million reads were generated and preprocessed with Trimmomatic v. 0.32 (*leading: 3, trailing: 3, slidingwindow: 4:20, minlen: 75*; Trimmomatic, RRID:SCR_011848) [37]. These reads were then assembled *de novo* using Trinity v. r20140717 (Trinity, RRID:SCR_013048) [38], producing a 305-Mb transcriptome consisting of 321 855 transcripts.

## Genome Annotation

The MAKER2 genome annotation pipeline [39] predicted protein-coding genes using 3 approaches: (i) homology to fish proteins, (ii) assembled transcripts as RNA-seq evidence, and (iii) *de novo* gene predictors. Protein sequences from 11 other fish species on Ensemble and the set of Murray cod transcripts assembled in this study were aligned to the genome in a preliminary MAKER run as evidence to retrain *ab initio* gene predictors such as Augustus (Augustus: Gene Prediction, RRID:SCR_008417) [40] and SNAP [41]. These higher-quality gene models are then used in subsequent runs to predict the final set of Murray cod protein-coding genes. The pipeline identified 26 539 genes with an average annotation edit distance (AED) of 0.187 [42].

NCBI’s *blastp* (*-evalue 1e^−^^10^, -seg yes, -soft_masking true, -lcase_masking*, and hit fraction of ≥70% target length; BLASTP, RRID:SCR_001010) [43] was used to functionally annotate the gene sequences against vertebrate sequences in the NCBI non-redundant database, after which un-annotated sequences were searched against all sequences in the NCBI non-redundant database. Additional functional annotation was performed with InterProScan (InterProScan, RRID:SCR_005829) [44] to examine motifs, domains, and signatures in the Murray cod protein sequences based on information from public databases, including PANTHER (PANTHER, RRID:SCR_004869) [45], Pfam (Pfam, RRID:SCR_004726) [46], PRINTS (PRINTS, RRID:SCR_003412) [47], PROSITE (PROSITE, RRID:SCR_003457) [48], SMART (SMART, RRID:SCR_005026) [49], SUPERFAMILY (SUPERFAMILY, RRID:SCR_007952) [50], and TIGRFAMs (JCVI TIGRFAMS, RRID:SCR_005493) [51]. As a result, 96.5% of the predicted protein-coding genes were successfully annotated by at least 1 of the 2 methods (*blastp* 69%, InterProScan 96.1%).

## Conclusion

Having assembled and annotated the genome of an Australian teleost fish, we anticipate that this will be a catalyst for a range of genetic, genomic, and evolution-related studies of the Murray cod and related fish species (Harrisson et al., submitted for publication). In this study, we demonstrate that, despite its reported high error rate, low-coverage Nanopore long reads are still useful for scaffolding fish genome assembly. However, low-coverage long reads still pose limitations in (i) the full utilization of these reads, e.g., sequence self-correction and the use of long reads itself for assembly and gap-filling, and (ii) the choice of the most suitable assembly and scaffolder programs. Given the relative ease of generating Nanopore MinION reads and continuous improvement in data yield and read accuracy of this sequencing platform, we look forward to overcoming these limitations and to further incorporating Nanopore long read information into eurkaryote genome assemblies, either in hybrid approaches or, ideally and ultimately, in non-hybrid *de novo* assemblies. We anticipate that Nanopore long reads will increasingly complement or even supersede short read data for the *de novo* genome assembly of fish species.

## Availability of supporting data

The data sets supporting the results of this article are available in the *Giga*DB repository [52]. Raw reads (Illumina and Nanopore) are available in the Sequence Read Archive (SRA), and the Whole Genome Shotgun project has been deposited at DDBJ/EMBL/GenBank under accession number LKNJ00000000 (first version), both under BioProject PRJNA290988. Similarly, transcriptome (Illumina) reads are also available in the SRA, and the Transcriptome Shotgun Assembly project has been deposited under accession number GFMM00000000 (first version) as part of BioProject PRJNA383091.

## Competing interests

The authors declare that they have no competing interests.

## Supplementary Material

GIGA-D-17-00103_Original-Submission.pdf

GIGA-D-17-00103_Revision-1.pdf

GIGA-D-17-00103_Revision-2.pdf

Response-to-Reviewer-Comments_Original-Submission.pdf

Response-to-Reviewer-Comments_Revision-1.pdf

Reviewer-1-Report-(Original-Submission).pdf

Reviewer-1-Report-(Revision-1).pdf

Reviewer-2-Report-(Original-Submission).pdf

Reviewer-2-Report-(Revision-1).pdf

Supplement Materials
