# Supplementary material for: De novo genome assembly and annotation of Australia's largest freshwater fish, the Murray cod (Maccullochella peelii), from Illumina and Nanopore sequencing read
Source: Gigascience. 2017 Jul 19;6(8):1–6. doi: 10.1093/gigascience/gix063 (PMC5597895; doi:10.1093/gigascience/gix063)
Supplement: GIGA-D-17-00103_Original-Submission.pdf [file gix063_GIGA-D-17-00103_Original-Submission.pdf]

Manuscript
Click here to download Manuscript MurrayCod_GigaScience_040517.doc

The long and the short of it: Low coverage Nanopore reads significantly improved the contiguity of Illumina-based genome assembly of Australia's largest freshwater fish, the Murray cod (*Maccullochella peelii*)

Christopher M. Austin[1,2,3#], Mun Hua Tan[1,2,3#], Katherine A. Harrisson[4], Yin Peng Lee[2,3], Laurence J. Croft[3,5], Paul Sunnucks[4], Alexandra Pavlova[4], Han Ming Gan[1,2,3]*

[1] School of Life and Environmental Sciences, Deakin University, Geelong, Victoria 3216, Australia

[2] Genomics Facility, Tropical Medicine and Biology Platform, Monash University Malaysia, Jalan Lagoon Selatan, Bandar Sunway 47500, Petaling Jaya, Selangor, Malaysia

[3] School of Science, Monash University Malaysia, Jalan Lagoon Selatan, Bandar Sunway 47500, Petaling Jaya, Selangor, Malaysia

[4] School of Biological Sciences, Monash University, Clayton Campus, Clayton, Victoria, Australia

[5] Malaysian Genomics Resource Centre Berhad, Boulevard Signature Office, Kuala Lumpur, Malaysia

[#] Equal contribution

*** Corresponding author:**

Name: Han Ming Gan, PhD

Address: Building 3, Level 3, Room 3-3-20, School of Science, Monash University Malaysia, Jalan Lagoon Selatan, Bandar Sunway, 47500, Petaling Jaya, Selangor, Malaysia

Phone: (+603) 5514 6000 (ext. 61727)

Email: gan.han.ming@monash.edu

Abstract

Background: One of the most iconic Australian fish is the Murray cod, *Maccullochella peelii* (Mitchell, 1838), a freshwater species that can grow to ~1.8 metres in length and live ≥ 48 years of age. The Murray cod is of conservation concern as a result of strong population contractions, but is also popular for recreational fishing and is of growing aquaculture interest. In this study, we report the whole genome sequence of the Murray cod to support ongoing population genetics, conservation and management-related research, as well as to understand better the evolutionary ecology and history of the species. Further, using a data set comprising high-coverage short and low-coverage long reads from the Illumina and Oxford Nanopore platforms respectively, we demonstrate that hybrid assembly is an efficient approach for generating high-quality reference genomes for fish species.

Findings: A draft Murray cod genome of 633 Mbp (N_{50}=109,974bp; CEGMA and BUSCO completeness of 91.94% and 89.4%, respectively) with an estimated 148 Mbp of putative repetitive sequences was assembled from 70 Gbp of data generated using the Illumina HiSeq and MiSeq platforms, with additional long reads from the Nanopore MinION sequencer at a low coverage (804 Mbp). Importantly, the inclusion of Nanopore reads led to a 29% reduction in the number of scaffolds and a 55% increase in the scaffold N50. We also report the first transcriptome of Murray cod that was subsequently used to accurately annotate the Murray cod genome leading to the identification of 26,539 protein-coding genes.

Conclusions: We present the first whole genome of an Australian teleost fish and anticipate this will be a catalyst for a range of genetic, genomic and phylogenetic

studies of the Murray cod and more generally other fish species of Percichthydae family. Further, we recommend combining low to medium coverage MinION Nanopore long reads with short read assemblies as a practical and effective approach to generating more contiguous fish genomes assemblies.

Keywords: Murray Cod, long reads, genome, transcriptome, hybrid assembly

Data description

Population genetic and evolutionary studies on Australian freshwater fish are of special interest in relation to conservation, biogeography and adaptive responses and have been studied using a range of molecular techniques [1-8] (Harrisson et al, submitted). A limitation to a more complete understanding of the genetics and evolution of Australian inland fish species and the ability to undertake a number of different kinds of related studies is the lack of genome level resources. The Murray cod is one of Australia's most iconic large (up to ~1.8 metres) and long-lived (≥ 48 years) predatory fish species that occurs across highly variable and heterogeneous riverine environments of inland Australia. Despite being widespread, the Murray cod is a threatened species under national legislation (Environment Protection and Biodiversity Conservation Act 1999) and populations are intensively managed through programs such as habitat restoration, provision of environmental flows and stocking.

Sampling, library construction and sequencing

Sequencing data from two Murray cod individuals were merged for whole genome assembly. The first individual was obtained from an Australian fish market in 2014

[5]. Genomic DNA was extracted from multiple fin clip and muscle samples using DNAeasy Blood and Tissue Kits (Qiagen, Halden, Germany). A 300 bp insert library was prepared from the purified gDNA using TruSeq DNA sample prep kit (Illumina, San Diego, CA) according to the manufacturer's instructions and subsequently sequenced (2 × 100 bp configuration) on a HiSeq 2000 (Illumina, San Diego, CA) located at the Malaysian Genomics Resource Centre Berhad. For sequencing on the MinION, gDNA was extracted from the remaining fin clip and muscle tissues collected in year 2014. However, due to DNA degradation associated with long-term storage, an additional size selection (8 – 30 kb) with a BluePippin was performed to reduce the representation of short reads (Sage Science, Beverly, MA). Seven individual libraries (two 1D preps and five 2D preps) were prepared and sequenced on seven R9 flowcells using the MinION portable DNA sequencer (Oxford Nanopore, UK) according to the manufacturer's instructions. The second Murray cod, isolate KMC200, was sampled from the Lachlan River in New South Wales in 2006 and has its library previously constructed and sequenced at the Monash University Malaysia Genomics Facility for a mitogenome-based population genetics study [2, 5]. Given that isolate KMC200 exhibit a 100% whole mitogenome nucleotide identity to that of the first individual [2, 5], its remaining library was re-sequenced in three separate MiSeq runs (2 × 250 bp configuration). A total of 70.6 Gb (47.4 Gb and 23.2 Gb from HiSeq and MiSeq runs, respectively) and 804 Mb (N_{50}: 4,438 bp, longest read: 129,945 bp) of nucleotide sequence were generated on the Illumina platforms and the Oxford Nanopore MinION device respectively.

Genome characteristics

Jellyfish v.2.2.6 [9] was used to obtain a frequency distribution of 17-, 21-, 25- and

31-mers in raw HiSeq sequence reads and the histograms were uploaded to GenomeScope for estimation of genome size, repeat content and heterozygosity, based on a kmer-based statistical approach [10]. The resulting analysis shows that the haploid genome size was between 640 to 669 Mbp for the Murray cod (Figure 1), a figure smaller than the 812 Mbp (C-value: 0.83 pg) estimated size reported on the Animal Genome Size Database [11, 12]. This smaller estimate may be due to an additional parameter introduced in GenomeScope, set to exclude extremely high frequency kmers as these likely represent organelle sequences or other contaminants that can inflate the genome size [10]. Further, the 21-mer analysis (with *max kmer coverage* set at 1000) on GenomeScope also indicates 14.3% repeat content and low heterozygosity of 0.103%. Further repeat-content analysis and masking is performed in subsequent sections in this study (see ‘Repeat-content analysis’).

Genome assembly

Illumina reads were trimmed with *platanus_trim* v.1.0.7 (-*q* 20, -*l* 35) and assembled with the Platanus v.1.2.4 assembler to account for increase in genome heterozygosity due to the use of sequencing data from two individuals with shared maternal lineage [13]. The initial assembly is 622 Mb in length, comprising 80,098 scaffolds with an N50 of 68,937 bp (Table 1). The assembly was subsequently scaffolded with SSPACE-LongRead v.1-1 [14] using long-read MinION sequences (0.93× coverage), which was base-called offline with Albacore/ONT Sequencing Pipeline Software v.0.7.4 followed by further gap-filling with Illumina reads using GapFiller v.1-10 [15]. By adding only 804 Mb of long reads, we observed significant improvements in the contiguity of the final 633 Mb assembly, reducing the number of scaffolds (≥ 500bp) by 29% from 25,642 to 18,198 and increasing the scaffold N50 by 55% from

70,993 bp to 109,975 bp. Finally, CEGMA v.2.5 [16] suggests a high level of genome completeness with almost 92% of the 248 conserved core eukaryotic genes (CEGs) identified. The genome completeness of the final genome assembly was also estimated using BUSCO, which searched the predicted proteome against a more comprehensive and fish-specific ortholog database (Actinoptergyii_odb9, creation date: 13th February 2016) that was constructed from 20 fish species and consists of 4,584 orthologs. BUSCO analysis estimated a genome completeness of 89.4% (86.0% complete and single copy; 3.4% complete and duplicated, Table 1) for the final annotated assembly, rising to 96.3% when fragmented/incomplete protein hits (6.9%) are included.

Repeat-content analysis

To identify repeats in the assembly, a *de novo* repeat library was first built with RepeatModeler v.1.0.4 [17] using default parameters based on the larger scaffolds (≥ 5kb) in the assembly. RepeatMasker v.open-4.0.7 [18] was then used to align sequences from the whole assembly to the RepeatMasker Combined Library (Dfam_Consensus 20170127 [19] and RepBase 20170127 [20]) as well as the *de novo* repeat library to screen for repeats and low complexity sequences in the assembly. Repeat sequences were estimated to account for 23.38% (148 Mb) of the Murray cod assembly presented in this study.

Transcriptome assembly

Total RNA was extracted using RiboPure RNA purification Kit (Thermo Fisher Scientific, Waltham, MA) from the liver, brain and muscle tissues of a juvenile Murray cod that was collected from a natural population in Broken Creek under a

DELWP collecting permit and euthanized using approved procedures under Monash ethics permit (BSCI/2012/19). Thirty μL of 300 ng/μL of each RNA extract was pooled and processed as a single sample using the TruSeq RNA library kit (Illumina, San Diego, CA) to generate a 160 bp insert size library. The library was subsequently sequenced on one lane of HiSeq2000 (2×100 bp configuration) at the Ramaciotti Centre for Gene Function Analysis. A total of 376 million reads was generated and preprocessed with Trimmomatic v.0.32 (*leading: 3, trailing: 3, slidingwindow:4:20, minlen:75*) [21]. These reads were then assembled *de novo* using Trinity v. r20140717 [22], producing a 305 Mb transcriptome of 321,855 transcripts.

Genome annotation

The MAKER2 genome annotation pipeline [23] predicted protein-coding genes using three approaches: 1) homology to fish proteins, 2) Murray cod RNA-seq evidence and 3) *de novo* gene predictors. Protein sequences from 11 other fish species on Ensemble and the set of Murray cod transcripts assembled in this study were aligned to the genome in a preliminary MAKER run as evidence to retrain *ab initio* gene predictors such as Augustus [24] and SNAP [25]. These higher-quality gene models are then used in subsequent runs to predict the final set of Murray cod protein-coding genes. The pipeline identified 26,539 genes with an average Annotation Edit Distance (AED) of 0.187 [26].

NCBI's *blastp* (*-evalue* $1e^{-10}$, *-seg yes, -soft_masking true, -lcase_masking,* and hit fraction of ≥70 % target length) [27] was used to functionally annotate the gene sequences against vertebrate sequences in the NR database, after which, un-annotated sequences were searched against all sequences in the NR database. Additional functional annotation was performed with InterProScan [28] to examine

motifs, domains and signatures in the Murray cod protein sequences based on information from public databases including PANTHER [29], Pfam [30], PRINTS [31], PROSITE [32], SMART [33], SUPERFAMILY [34] and TIGRFAMs [35]. As a result, 96.5% of the predicted protein-coding genes were successfully annotated by at least one of the two methods (*blastp* 69%, InterProScan 96.1%).

Conclusion

Having generated, assembled and successfully annotated the first genome of an Australian teleost fish, we anticipate this will be a catalyst for a range of genetic, genomic and evolution-related studies of the Murray cod and related species (Harrisson et al, submitted). Further, we recommend a hybrid approach using a combination of sequencing reads derived from Illumina and Nanopore platforms as a practical and effective approach to generating fish genome assemblies.

Availability of supporting data

The data sets supporting the results of this article are available in the GigaDB repository. Raw genome reads (Illumina and Nanopore) are available in the Sequence Read Archive (SRA) and the Whole Genome Shotgun project has been deposited at DDBJ/EMBL/GenBank under the accession LKNJ00000000 (first version), both under BioProject PRJNA290988. Similarly, transcriptome (Illumina) reads are also available in the SRA and the Transcriptome Shotgun Assembly project has been deposited under the accession GFMM00000000 (first version) as part of BioProject PRJNA383091.

Acknowledgements

This study was funded by Monash University Malaysia Tropical and Biology Multidisciplinary Platform and ARC grant LP110200017 to Monash University, Flinders University, and the University of Canberra, and Partner Organization University of Montana, with Partner Organizations ACTEW Corporation, Department of Sustainability and Environment (Victoria) (now Department of Environment, Land, Water & Planning, DELWP), Fisheries Victoria (now within Department of Economic Development, Jobs, Transport and Resources), and Melbourne Water. We thank Joanne Kearns and Jarod Lyon from Arthur Rylah Institute (DELWP) and Dean Gilligan and Meaghan Rourke from NSW Department of Primary Industries (NSW DPI) for assistance in sample collection, Catriona Millen for assistance with RNA extraction, and Steven Amish for assistance with preliminary transcriptome assembly. We also acknowledge the Monash University Malaysia High Performance Computing infrastructure for computational resources.

Competing interests

The authors declade that they have no competing interests

Figure

Figure 1. Estimation of genome size, repeat content and heterozygosity by GenomeScope, based on 21-mers in HiSeq sequence reads (*max kmer coverage* at 1000).

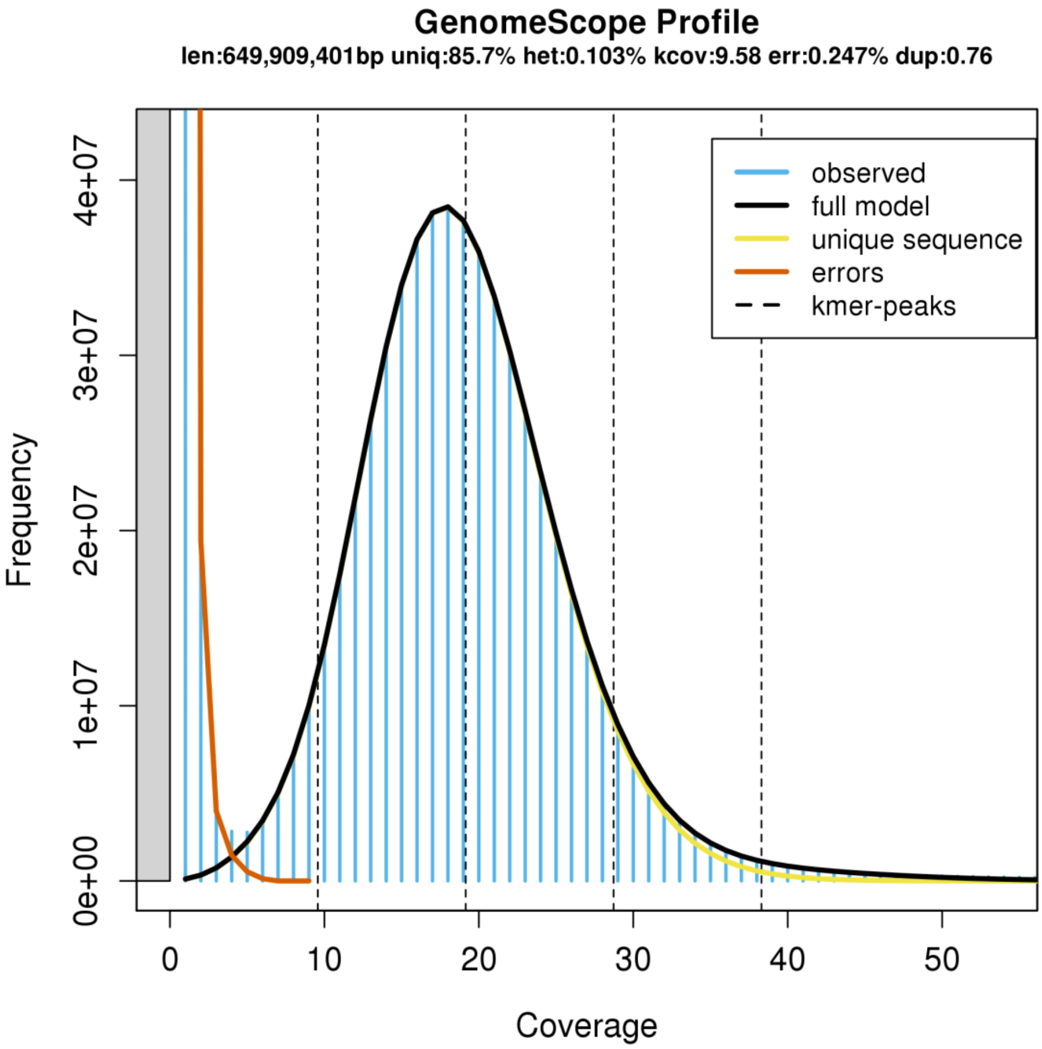

Table

Table 1. Murray cod assembly and annotation statistics.

**Genome Assembly**	**Illumina only**	**Illumina (≥500bp)**	**Illumina + Nanopore**
Number of scaffolds	80,098	25,642	18,198
Total scaffold size	622,421,194 bp	609,090,121 bp	633,241,041 bp
Scaffold N50 size	68,937 bp	70,993 bp	109,974 bp
Shortest scaffold	174 bp	500 bp	500 bp
Longest scaffold	548,726 bp	548,726 bp	1,119,190 bp
% GC / AT / N	40.7 / 59.1 / 0.2	40.7 / 59.2 / 0.1	40.4 / 58.7 / 0.9
CEGMA completeness	89.52%	84.68%	91.94%
Complete BUSCOs			4,095 (89.4%)
Complete and single-copy BUSCOs			3,941 (86.0%)
Complete and duplicated BUSCOs			154 (3.4%)
Fragmented BUSCOs			318 (6.9%)
Missing BUSCOs			171 (3.7%)
Total BUSCO groups searched			4,584

**Transcriptome Assembly**	
Number of transcripts	321,855
Transcriptome size	305,149,376 bp
Mean transcript length	948.10 bp
Longest transcript	23,655 bp
CEGMA completeness	99.19%
**Annotation**	
Number of protein-coding genes	26,539
Mean gene length	10115.3 bp
Longest gene	134,909 bp
With functional annotation	25,607

References

1. Pavlova, A., et al., *Purifying selection and genetic drift shaped Pleistocene evolution of the mitochondrial genome in an endangered Australian freshwater fish.* Heredity, 2017.
2. Harrisson, K., et al., *Pleistocene divergence across a mountain range and the influence of selection on mitogenome evolution in threatened Australian freshwater cod species.* Heredity, 2016. **116**(6): p. 506-515.
3. Cole, T.L., et al., *Range-wide fragmentation in a threatened fish associated with post-European settlement modification in the Murray–Darling Basin, Australia.* Conservation Genetics, 2016. **17**(6): p. 1377-1391.
4. Unmack, P.J., et al., *Genome-wide SNPs resolve a key conflict between sequence and allozyme data to confirm another threatened candidate species of river blackfishes (Teleostei: Percichthyidae: Gadopsis).* Molecular Phylogenetics and Evolution, 2017. **109**: p. 415-420.
5. Austin, C.M., et al., *The complete mitogenome of the Murray Cod, Maccullochella peelii (Mitchell, 1838) (Teleostei: Percichthyidae).* Mitochondrial DNA Part A, 2016. **27**(1): p. 729-730.
6. Harrisson, K.A., et al., *Identifying environmental correlates of intraspecific genetic variation.* Heredity, 2016. **117**(3): p. 155-164.
7. Pavlova, A., et al., *Severe consequences of habitat fragmentation on genetic diversity of an endangered Australian freshwater fish: a call for assisted gene flow.* Evolutionary Applications: p. n/a-n/a.
8. Hermoso, V., et al., *Species distributions represent intraspecific genetic diversity of freshwater fish in conservation assessments.* Freshwater Biology, 2016. **61**(10): p. 1707-1719.
9. Marçais, G. and C. Kingsford, *A fast, lock-free approach for efficient parallel counting of occurrences of k-mers.* Bioinformatics, 2011. **27**(6): p. 764-770.
10. Vurture, G.W., et al., *GenomeScope: Fast reference-free genome profiling from short reads.* bioRxiv, 2016.
11. Gregory, T.R. *Animal Genome Size Database*. 2017; Available from: http://www.genomesize.com.
12. Hardie, D.C. and P.D.N. Hebert, *Genome-size evolution in fishes.* Canadian Journal of Fisheries and Aquatic Sciences, 2004. **61**(9): p. 1636-1646.
13. Kajitani, R., et al., *Efficient de novo assembly of highly heterozygous genomes from whole-genome shotgun short reads.* Genome Research, 2014.
14. Boetzer, M. and W. Pirovano, *SSPACE-LongRead: scaffolding bacterial draft genomes using long read sequence information.* BMC Bioinformatics, 2014. **15**(1): p. 211.
15. Boetzer, M. and W. Pirovano, *Toward almost closed genomes with GapFiller.* Genome Biology, 2012. **13**(6): p. R56.
16. Parra, G., K. Bradnam, and I. Korf, *CEGMA: a pipeline to accurately annotate core genes in eukaryotic genomes.* Bioinformatics, 2007. **23**(9): p. 1061-1067.
17. Smit, A., Hubley, R. *RepeatModeler Open-1.0*. 2008-2015; Available from: http://www.repeatmasker.org.
18. Smit, A., Hubley, R & Green, P. *RepeatMasker Open-4.0*. 2013-2015; Available from: http://www.repeatmasker.org.
19. Hubley, R., et al., *The Dfam database of repetitive DNA families.* Nucleic Acids Research, 2016. **44**(D1): p. D81-D89.

20. Jurka, J., et al., *Repbase Update, a database of eukaryotic repetitive elements.* Cytogenetic and Genome Research, 2005. **110**(1-4): p. 462-467.
21. Bolger, A.M., M. Lohse, and B. Usadel, *Trimmomatic: a flexible trimmer for Illumina sequence data.* Bioinformatics, 2014. **30**(15): p. 2114-2120.
22. Grabherr, M.G., et al., *Full-length transcriptome assembly from RNA-Seq data without a reference genome.* Nat Biotech, 2011. **29**(7): p. 644-652.
23. Holt, C. and M. Yandell, *MAKER2: an annotation pipeline and genome-database management tool for second-generation genome projects.* BMC Bioinformatics, 2011. **12**(1): p. 491.
24. Stanke, M., et al., *Gene prediction in eukaryotes with a generalized hidden Markov model that uses hints from external sources.* BMC Bioinformatics, 2006. **7**(1): p. 62.
25. Korf, I., *SNAP: Semi-HMM-based Nucleic Acid Parser.* Ian Korf homepage: http://homepage. mac. com/iankorf, 2013.
26. Eilbeck, K., et al., *Quantitative measures for the management and comparison of annotated genomes.* BMC Bioinformatics, 2009. **10**(1): p. 67.
27. Altschul, S.F., et al., *Basic local alignment search tool.* Journal of Molecular Biology, 1990. **215**(3): p. 403-410.
28. Jones, P., et al., *InterProScan 5: genome-scale protein function classification.* Bioinformatics, 2014. **30**(9): p. 1236-1240.
29. Mi, H., A. Muruganujan, and P.D. Thomas, *PANTHER in 2013: modeling the evolution of gene function, and other gene attributes, in the context of phylogenetic trees.* Nucleic Acids Research, 2013. **41**(Database issue): p. D377-D386.
30. Punta, M., et al., *The Pfam protein families database.* Nucleic Acids Research, 2012. **40**(Database issue): p. D290-D301.
31. Attwood, T.K., et al., *The PRINTS database: a fine-grained protein sequence annotation and analysis resource—its status in 2012.* Database: The Journal of Biological Databases and Curation, 2012. **2012**: p. bas019.
32. Sigrist, C.J.A., et al., *New and continuing developments at PROSITE.* Nucleic Acids Research, 2013. **41**(Database issue): p. D344-D347.
33. Letunic, I., T. Doerks, and P. Bork, *SMART 7: recent updates to the protein domain annotation resource.* Nucleic Acids Research, 2012. **40**(Database issue): p. D302-D305.
34. de Lima Morais, D.A., et al., *SUPERFAMILY 1.75 including a domain-centric gene ontology method.* Nucleic Acids Research, 2011. **39**(Database issue): p. D427-D434.
35. Haft, D.H., et al., *TIGRFAMs and Genome Properties in 2013.* Nucleic Acids Research, 2013. **41**(D1): p. D387-D395.
