# Supplementary material for: De novo genome assembly and annotation of Australia's largest freshwater fish, the Murray cod (Maccullochella peelii), from Illumina and Nanopore sequencing read
Source: Gigascience. 2017 Jul 19;6(8):1–6. doi: 10.1093/gigascience/gix063 (PMC5597895; doi:10.1093/gigascience/gix063)
Supplement: Supplement Materials [file gix063_Supp.zip › Supplementary Data 1.pdf]

Scaffolds specific to each donor:

MiSeq-only scaffolds	Scaffold length (bp)	HiSeq-only scaffolds	Scaffold length (bp)
LKNJ01011177.1	2714	LKNJ01014528.1	726
LKNJ01011802.1	2039	LKNJ01014797.1	680
LKNJ01012063.1	1797	LKNJ01015632.1	548
LKNJ01012130.1	1750		
LKNJ01012695.1	1377		
LKNJ01012790.1	1330		
LKNJ01012865.1	1283		
LKNJ01012919.1	1258		
LKNJ01012978.1	1228		
LKNJ01012986.1	1226		
LKNJ01013115.1	1163		
LKNJ01013635.1	974		
LKNJ01013764.1	931		
LKNJ01014103.1	829		
LKNJ01014179.1	813		
LKNJ01014191.1	968		
LKNJ01014294.1	784		
LKNJ01014313.1	780		
LKNJ01014763.1	601		
LKNJ01014788.1	622		
LKNJ01014838.1	672		
LKNJ01014862.1	667		
LKNJ01015099.1	573		
LKNJ01015135.1	619		
LKNJ01015149.1	616		
LKNJ01015385.1	578		
LKNJ01015427.1	573		
LKNJ01015448.1	570		
LKNJ01015588.1	553		
LKNJ01015609.1	550		
LKNJ01015701.1	539		
LKNJ01015806.1	526		
LKNJ01015874.1	519		
LKNJ01015996.1	502		
LKNJ01017076.1	511		
LKNJ01017411.1	926		
LKNJ01017978.1	574		
