# Supplementary figures and images for: De novo genome assembly and annotation of Australia's largest freshwater fish, the Murray cod (Maccullochella peelii), from Illumina and Nanopore sequencing read
Source: Gigascience. 2017 Jul 19;6(8):1–6. doi: 10.1093/gigascience/gix063 (PMC5597895; doi:10.1093/gigascience/gix063)

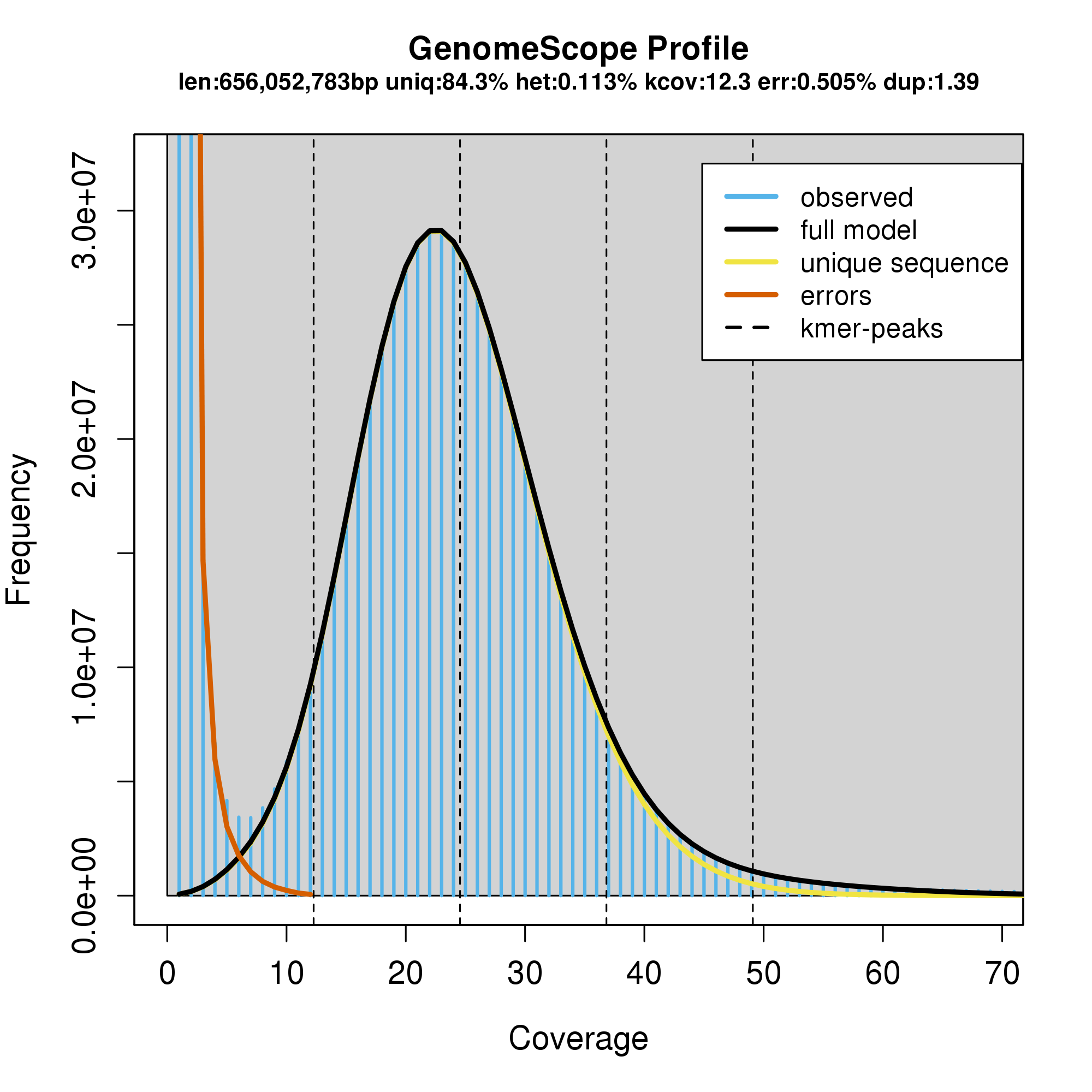

Supplement: Supplement Materials [file gix063_Supp.zip › Supplementary_Figure_1.png]

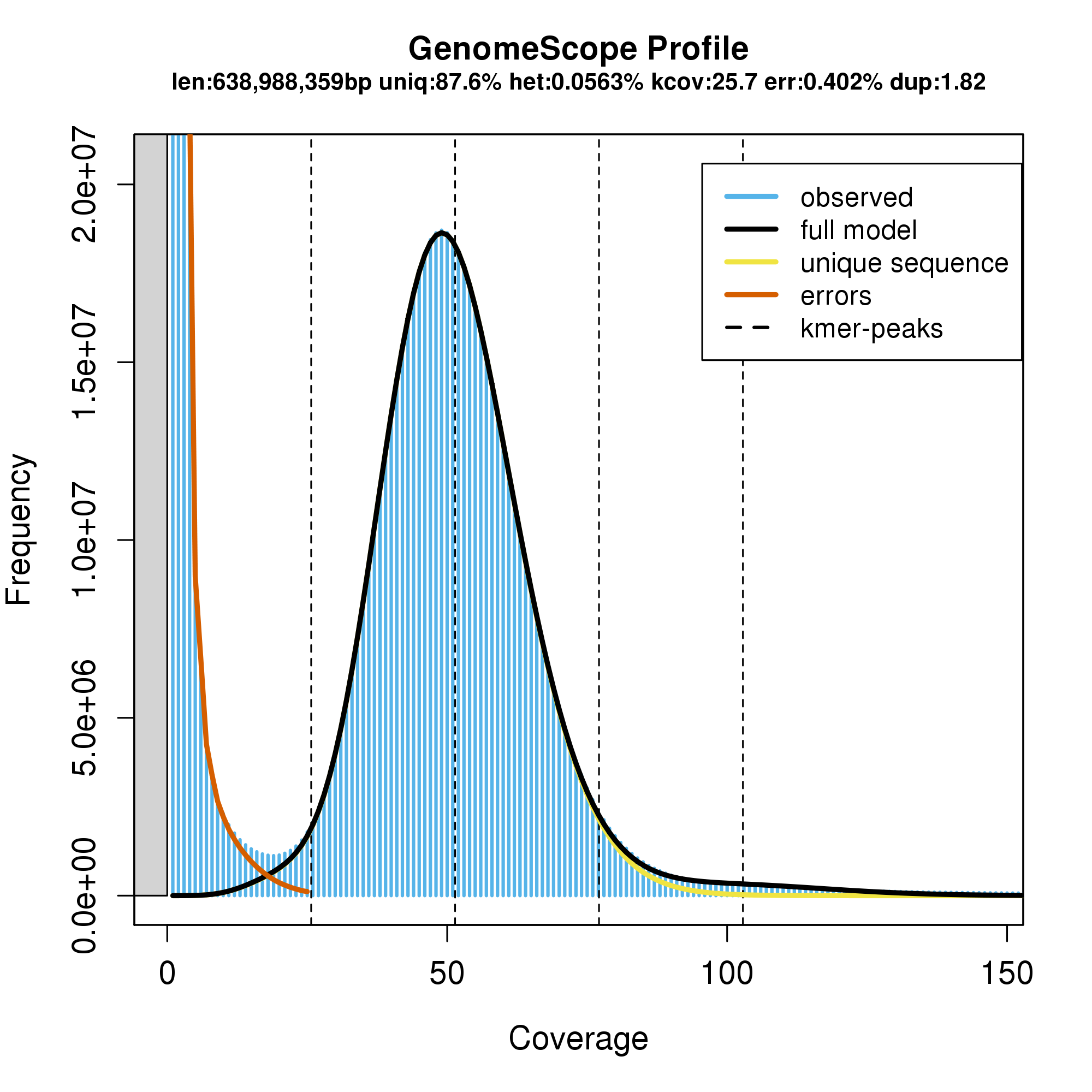

Supplement: Supplement Materials [file gix063_Supp.zip › Supplementary_Figure_2.png]
