# Supplementary material for: De novo genome assembly and annotation of Australia's largest freshwater fish, the Murray cod (Maccullochella peelii), from Illumina and Nanopore sequencing read
Source: Gigascience. 2017 Jul 19;6(8):1–6. doi: 10.1093/gigascience/gix063 (PMC5597895; doi:10.1093/gigascience/gix063)
Supplement: Response-to-Reviewer-Comments_Original-Submission.pdf [file gix063_Response-to-Reviewer-Comments_Original-Submission.pdf]

Author's Response To Reviewer Comments

Reviewer #1
Comment 1:
This paper describes the sequencing and assembly methodology, as well as the annotation of the Murray cod genome. The data and methods used are appropriate, and the genome assembly and annotation are of acceptable quality. The contiguity is modest, as only limited scaffolding was performed. Since the scaffolding procedure is presented as the major message of the paper (both in the title and the conclusion), I would like to ask the authors to elaborate on this topic:

1 SSPACE-LongRead was used for scaffolding. This choice needs justification and additional details (settings), as this application was originally designed for bacterial genomes and (high coverage) PacBio data. To my knowledge, there have been no updates to make it suitable for Oxford Nanopore data. Did you use the default aligner (BLASR) and default alignment settings? Was a single read link considered sufficient for contig linking? Does the assumption of a bacterial genome (high sequence complexity, few contigs) affect scaffolding of a large animal genome (lower sequence complexity, many contigs)?

Response:
Following comments from both reviewers, we have made edits to the title and conclusion to better align with the topic of the manuscript and study.
We have also added our justification for using SSPACE-LongRead at lines 164 to 179 as well as described settings used in this study is added at lines 148 to 149.

Comment 2:
2 The CEGMA results (Table 1) suggest the scaffolding procedure makes correct choices. Could you also provide a BUSCO analysis for the input dataset (i.e. Illumina >500 bp)?

Response:
We have added BUSCO analysis results to Table 1 for both Illumina assemblies (with and without the >500 bp length cutoff).

Comment 3:
3 The authors recommend an Illumina de novo assembly plus low coverage nanopore scaffolding as a 'practical and effective approach' (line 211-213). Could you elaborate on this, perhaps with some suggestions for future directions? For example, one thing that I would like to see implemented in such an approach is the use of nanopore data in gap filling. Currently, long-read data is only used for structural information, and scaffolding leads to gaps. These are then later addressed using GapFiller, but wouldn't it be better to somehow salvage the long-read sequence information and use this as a template for gap filling/sequence correction? Also, how will the increasing yields of the ONT platform affect future scaffolding approaches?

Response:
We agree that there is a lot more potential to long reads than only using them for structural information, especially for large eukaryotic genomes. However, in this study specifically, our nanopore data was from earlier versions of the technology with relatively higher error rates and

lower data yield. Because of these limitations, we chose the more conservative approach of using long reads only for scaffolding than to incorporate it into the assembly itself.

We have added mention of the limitations of using low coverage nanopore reads at lines 164 to 165 and 231 to 239. Given the rapid recent advances of the nanopore technology in terms of sequence quality and yield, we also listed some assembly/scaffolding tools that have been made available recently (lines 177 to 179) as alternatives that can be explored to more fully utilize the potential of long reads.

Comment 4:

Further minor comments:

4 The assembly is based on two individuals. Overall, the heterozygosity appears to be low (Figure 1), which justifies this approach. However, this analysis is based on only one of the individuals (the fish market one). This, and the fact that only a subset of the HiSeq data have been used for this analysis, could be stated more clearly. In addition, a similar analysis for the other fish (MiSeq data) would be appropriate.

Response:
We have repeated the heterozygosity estimation analysis for the other fish (MiSeq data) at lines 134 to 138 and included it as Supplementary Figure 1.

Comment 5:

5 The two individuals have a 'shared maternal lineage' (line 142). How has this been determined?

Response:
This was determined based on the 100% nucleotide sequence identity between the two mitochondrial genomes available on GenBank (Accession numbers: NC_023807.1 [Individual 1]and KT337332.1 [Individual 2])

Comment 6:

6 The de novo transcriptome assembly produces and oversized transcriptome (line 184). Please comment briefly on the discrepancy between the de novo transcriptome assembly and the apparent transcriptome size from genome annotation.
Is this due to the mixed sample used (line 174)? Were these de novo transcripts used as annotation evidence, or was this done using read alignment (line 188)

Response:
The discrepancy may be due to the multiple sources of tissue samples that were pooled and sequenced at very high sequencing depth, resulting in isoforms and alternate splice variants expressed by the different tissues. We did not perform further steps to reduce redundancy in the assembly as our aim is mainly to use these transcripts as evidence for the genome annotation, not for any sort of expression/DE analysis or investigation of core genes among different tissue

types. These de novo transcripts were provided to the MAKER gene prediction program as annotation evidence. On the contrary, in this study, the transcripts reported by MAKER from the genome are the longest isoforms as single representatives for each gene. The number of protein-coding genes predicted from the Murray cod appears within the range of what has been predicted for most other fish genomes (e.g. Species - #coding genes: Amazon molly – 23615, Cave fish – 23042, Cod – 20095, Fugu – 18523, Medaka – 19699, Platyfish – 20379, Spotted gar – 18341, Stickleback – 20787, Tetraodon – 19602, Tilapia – 21437, Zebrafish – 25832, Arowana – 24274, Salmon - 37206)

___

Reviewer #2
Comment 1:
Using two donors, the fish genome was sequenced and assembled, quantified for repeats, and gene-annotated with new and existing transcript sequence. Most of the process used standard practices and overall it appears well done. The genome and transcript assemblies are in GenBank. A new long-read technology was incorporated to extend the assembly scaffolds. The result was validated by gene content but no other means.

I found the title misleading. The Title implies that the manuscript is mostly about integrating long and short reads. The Abstract is also largely about Nanopore. With this setup, the manuscript is bound to disappoint: no comparison of Nanopore to other long-range linkage technologies, no titration analysis of benefit per unit coverage, no examination of whether the 2D reads helped more than the 1D reads, no comparison of Nanopore basecallers, no quantification of read accuracy, no analysis of whether the scaffold software performed correctly on data for which it was not designed, and most critically, no validation that the Nanopore extensions to scaffolds were correct.
* The manuscript will read better if the Title and Abstract change to reflect what the manuscript is actually about.
Response:
Following comments from both reviewers, we have made edits to the title and conclusion to further suit the topic of the manuscript and study.

___

Comment 2:
* The manuscript should address the unknown accuracy of the scaffold extensions.
Response:
The lack of verification of accuracy of scaffold extensions in this study is acknowledged at lines 175 to 177.

___

Comment 3:
* The words "practical and effective" (Conclusion) are not defined or backed by analysis.
Response:
We have made changes to the conclusion at lines 226 to 241.

___

Comment 4:
* The scaffold growth should not be called "significant" without statistics.
Response:

We have replaced the term with “substantial” in the manuscript (line 153).

Comment 5:
* For clarity, the word "contiguity" should be reserved for contig growth and not applied to scaffold growth.
Response:
We have removed the mention of “contiguity” in the manuscript.

Comment 6:
* The Nanopore read length N50 seems low but I don't know if there is anything to say about this.
Response:
This was the case because sequencing on the Nanopore was done much later after Illumina sequencing with degraded Murray cod gDNA as the only option.

Comment 7:

I found the use of a second donor to be problematic and it only struck me on the second read. The second donor may have introduced sequence differences and structural variations relative to the first. Different sequencing technologies were applied to each donor and the longer Illumina reads from the second donor could have biased the assembly toward it. The assembly could contain falsely duplicated genes that are single-copy in both donors but different enough to assemble separately. On the other hand, there may have been too few second-donor reads to matter, but then why were they used?
* The manuscript will be more helpful if this problem is addressed. Mention the dual donors in the Abstract. Elsewhere, explain why two donors were used. Quantify coverage per donor. Address whether the donors are the same gender.
Response:
The use of dual donors for the assembly is mentioned in the Abstract at lines 63 to 64 and in the text at lines 94 to 120. Based on read alignment results, an approximate average 56× coverage per site was calculated for HiSeq donor and average 25× coverage per site for MiSeq donor. Unfortunately, the gender of both fishes could not be determined as tissue was sampled from the fin.

Comment 8:
* Explore the possible effects of use of the second donor. Are any scaffolds formed from the reads of one donor only?
Response:
Based on read alignment results, only a minimal number of scaffolds and small percentage of the assembly were formed from reads of one donor only. 3 scaffolds (1,954 bp ~ 0.0003% genome fraction) with no coverage from HiSeq donor and 37 scaffolds (34,535 bp ~0.005% genome fraction) with no coverage from MiSeq donor.

Comment 9:
* Explain the use of Platanus. It seems this assembler was chosen to overcome heterozygosity, but what did this assembler do especially for the heterozygosity? The observed heterozygosity

(Figure 1) seems low, so perhaps the issue turned out less serious than anticipated. If so, was the use of Platanus risky in any way?

Response:

For our work, we also explored the use of MaSuRCA for our dataset. However, we found better results with Platanus for our dataset specifically in terms of number of scaffolds, scaffold N50, and CEGMA completeness results. As for the use of Platanus on genomes with low heterozygosity, the developers have demonstrated in their publication (https://www.ncbi.nlm.nih.gov/pmc/articles/PMC4120091) that the program is not restricted to highly heterozygous samples as it managed to produce the best validation results when tested on C. elegans data (0.0%-0.3% heterozygosity).

Comment 10:

The Abstract, Introduction, and Conclusion claim this is the first genome and transcriptome of an Australian teleost. The Data Description claims that a lack of similar resources limits current understanding. These claims beg for a reference. Failing that, the authors could describe how they came to these conclusions. Or they could save it for Discussion. Or use the dreaded "to our knowledge." I would leave it out.

Response:

We have removed claim of this being the first genome of an Australian teleost from the manuscript.
