# Supplementary material for: De novo genome assembly and annotation of Australia's largest freshwater fish, the Murray cod (Maccullochella peelii), from Illumina and Nanopore sequencing read
Source: Gigascience. 2017 Jul 19;6(8):1–6. doi: 10.1093/gigascience/gix063 (PMC5597895; doi:10.1093/gigascience/gix063)
Supplement: Response-to-Reviewer-Comments_Revision-1.pdf [file gix063_Response-to-Reviewer-Comments_Revision-1.pdf]

Author's Response To Reviewer Comments

Reviewer #2

Comment 1: The author’s revisions addressed my concerns.
The manuscript now contains a GenomeScope plot for each donor. It would make lots of sense to also include a 3rd plot for both donors combined. This makes sense since reads from both donors were used in the assembly. Presumably the heterozygosity would increase by a small amount.

Response:
We have included a GenomeScope plot as Supplementary Figure 2 to represent data combined from both donors as requested and showed that there was no substantial increase in heterozygosity that could have been caused from using two donors.
