# Supplementary material for: De novo genome assembly and annotation of Australia's largest freshwater fish, the Murray cod (Maccullochella peelii), from Illumina and Nanopore sequencing read
Source: Gigascience. 2017 Jul 19;6(8):1–6. doi: 10.1093/gigascience/gix063 (PMC5597895; doi:10.1093/gigascience/gix063)
Supplement: Reviewer-1-Report-(Original-Submission).pdf [file gix063_Reviewer-1-Report-(Original-Submission).pdf]

Reviewer Report

Title: De novo genome assembly and annotation of Australia's largest freshwater fish, the Murray cod (Maccullochella peelii) from Illumina and Nanopore sequencing reads

Version: Original Submission **Date:** 5/29/2017

Reviewer name: Christiaan Henkel

Reviewer Comments to Author:

This paper describes the sequencing and assembly methodology, as well as the annotation of the Murray cod genome. The data and methods used are appropriate, and the genome assembly and annotation are of acceptable quality. The contiguity is modest, as only limited scaffolding was performed. Since the scaffolding procedure is presented as the major message of the paper (both in the title and the conclusion), I would like to ask the authors to elaborate on this topic:1 SSPACE-LongRead was used for scaffolding. This choice needs justification and additional details (settings), as this application was originally designed for bacterial genomes and (high coverage) PacBio data. To my knowledge, there have been no updates to make it suitable for Oxford Nanopore data. Did you use the default aligner (BLASR) and default alignment settings? Was a single read link considered sufficient for contig linking? Does the assumption of a bacterial genome (high sequence complexity, few contigs) affect scaffolding of a large animal genome (lower sequence complexity, many contigs)?2 The CEGMA results (Table 1) suggest the scaffolding procedure makes correct choices. Could you also provide a BUSCO analysis for the input dataset (i.e. Illumina >500 bp)?3 The authors recommend an Illumina de novo assembly plus low coverage nanopore scaffolding as a 'practical and effective approach' (line 211-213). Could you elaborate on this, perhaps with some suggestions for future directions? For example, one thing that I would like to see implemented in such an approach is the use of nanopore data in gap filling. Currently, long-read data is only used for structural information, and scaffolding leads to gaps. These are then later addressed using GapFiller, but wouldn't it be better to somehow salvage the long-read sequence information and use this as a template for gap filling/sequence correction? Also, how will the increasing yields of the ONT platform affect future scaffolding approaches?Further minor comments:4 The assembly is based on two individuals. Overall, the heterozygosity appears to be low (Figure 1), which justifies this approach. However, this analysis is based on only one of the individuals (the fish market one). This, and the fact that only a subset of the HiSeq data have been used for this analysis, could be stated more clearly. In addition, a similar analysis for the other fish (MiSeq data) would be appropriate.5 The two individuals have a 'shared maternal lineage' (line 142). How has this been determined?6 The de novo transcriptome assembly produces and oversized transcriptome (line 184). Please comment briefly on the discrepancy between the de novo transcriptome assembly and the apparent transcriptome size from genome annotation.Is this due to the mixed sample used (line 174)? Were these de novo transcripts used as annotation evidence, or was this done using read alignment (line 188)

Level of Interest

Please indicate how interesting you found the manuscript: An article whose findings are important to those with closely related research interests

Quality of Written English

Please indicate the quality of language in the manuscript: Acceptable

Declaration of Competing Interests

Please complete a declaration of competing interests, considering the following questions:

- Have you in the past five years received reimbursements, fees, funding, or salary from an organisation that may in any way gain or lose financially from the publication of this manuscript, either now or in the future?
- Do you hold any stocks or shares in an organisation that may in any way gain or lose financially from the publication of this manuscript, either now or in the future?
- Do you hold or are you currently applying for any patents relating to the content of the manuscript?
- Have you received reimbursements, fees, funding, or salary from an organization that holds or has applied for patents relating to the content of the manuscript?
- Do you have any other financial competing interests?
- Do you have any non-financial competing interests in relation to this paper?

If you can answer no to all of the above, write 'I declare that I have no competing interests' below. If your reply is yes to any, please give details below.

I have received travel reimbursements from Oxford Nanopore Technologies.

I agree to the open peer review policy of the journal. I understand that my name will be included on my report to the authors and, if the manuscript is accepted for publication, my named report including any attachments I upload will be posted on the website along with the authors' responses. I agree for my report to be made available under an Open Access Creative Commons CC-BY license (http://creativecommons.org/licenses/by/4.0/). I understand that any comments which I do not wish to be included in my named report can be included as confidential comments to the editors, which will not be published.

I agree to the open peer review policy of the journal
