# Supplementary material for: De novo genome assembly and annotation of Australia's largest freshwater fish, the Murray cod (Maccullochella peelii), from Illumina and Nanopore sequencing read
Source: Gigascience. 2017 Jul 19;6(8):1–6. doi: 10.1093/gigascience/gix063 (PMC5597895; doi:10.1093/gigascience/gix063)
Supplement: Reviewer-2-Report-(Original-Submission).pdf [file gix063_Reviewer-2-Report-(Original-Submission).pdf]

Reviewer Report

Title: De novo genome assembly and annotation of Australia's largest freshwater fish, the Murray cod (Maccullochella peelii) from Illumina and Nanopore sequencing reads

Version: Original Submission **Date:** 5/31/2017

Reviewer name: Jason R. Miller, MS

Reviewer Comments to Author:

Using two donors, the fish genome was sequenced and assembled, quantified for repeats, and gene-annotated with new and existing transcript sequence. Most of the process used standard practices and overall it appears well done. The genome and transcript assemblies are in GenBank. A new long-read technology was incorporated to extend the assembly scaffolds. The result was validated by gene content but no other means.

I found the title misleading. The Title implies that the manuscript is mostly about integrating long and short reads. The Abstract is also largely about Nanopore. With this setup, the manuscript is bound to disappoint: no comparison of Nanopore to other long-range linkage technologies, no titration analysis of benefit per unit coverage, no examination of whether the 2D reads helped more than the 1D reads, no comparison of Nanopore basecallers, no quantification of read accuracy, no analysis of whether the scaffold software performed correctly on data for which it was not designed, and most critically, no validation that the Nanopore extensions to scaffolds were correct.

* The manuscript will read better if the Title and Abstract change to reflect what the manuscript is actually about.
* The manuscript should address the unknown accuracy of the scaffold extensions.
* The words "practical and effective" (Conclusion) are not defined or backed by analysis.
* The scaffold growth should not be called "significant" without statistics.
* For clarity, the word "contiguity" should be reserved for contig growth and not applied to scaffold growth.
* The Nanopore read length N50 seems low but I don't know if there is anything to say about this.

I found the use of a second donor to be problematic and it only struck me on the second read. The second donor may have introduced sequence differences and structural variations relative to the first. Different sequencing technologies were applied to each donor and the longer Illumina reads from the second donor could have biased the assembly toward it. The assembly could contain falsely duplicated genes that are single-copy in both donors but different enough to assemble separately. On the other hand, there may have been too few second-donor reads to matter, but then why were they used?

* The manuscript will be more helpful if this problem is addressed. Mention the dual donors in the Abstract. Elsewhere, explain why two donors were used. Quantify coverage per donor. Address whether

the donors are the same gender.
* Explore the possible effects of use of the second donor. Are any scaffolds formed from the reads of one donor only?
* Explain the use of Platanus. It seems this assembler was chosen to overcome heterozygosity, but what did this assembler do especially for the heterozygosity? The observed heterozygosity (Figure 1) seems low, so perhaps the issue turned out less serious than anticipated. If so, was the use of Platanus risky in any way?

The Abstract, Introduction, and Conclusion claim this is the first genome and transcriptome of an Australian teleost. The Data Description claims that a lack of similar resources limits current understanding. These claims beg for a reference. Failing that, the authors could describe how they came to these conclusions. Or they could save it for Discussion. Or use the dreaded "to our knowledge." I would leave it out.

Level of Interest

Please indicate how interesting you found the manuscript: An article whose findings are important to those with closely related research interests

Quality of Written English

Please indicate the quality of language in the manuscript: Acceptable

Declaration of Competing Interests

Please complete a declaration of competing interests, considering the following questions:

- Have you in the past five years received reimbursements, fees, funding, or salary from an organisation that may in any way gain or lose financially from the publication of this manuscript, either now or in the future?
- Do you hold any stocks or shares in an organisation that may in any way gain or lose financially from the publication of this manuscript, either now or in the future?
- Do you hold or are you currently applying for any patents relating to the content of the manuscript?
- Have you received reimbursements, fees, funding, or salary from an organization that holds or has applied for patents relating to the content of the manuscript?
- Do you have any other financial competing interests?
- Do you have any non-financial competing interests in relation to this paper?

If you can answer no to all of the above, write 'I declare that I have no competing interests' below. If your reply is yes to any, please give details below.

I declare that I have no competing interests

I agree to the open peer review policy of the journal. I understand that my name will be included on my report to the authors and, if the manuscript is accepted for publication, my named report including any attachments I upload will be posted on the website along with the authors' responses. I agree for my report to be made available under an Open Access Creative Commons CC-BY license (http://creativecommons.org/licenses/by/4.0/). I understand that any comments which I do not wish to be included in my named report can be included as confidential comments to the editors, which will not be published.

I agree to the open peer review policy of the journal
